# A scattered landscape: assessment of the evidence base for 71 patient decision aids developed in a hospital setting

**DOI:** 10.1186/s12911-022-01777-x

**Published:** 2022-02-17

**Authors:** Marion Danner, Marie Debrouwere, Anne Rummer, Kai Wehkamp, Jens Ulrich Rüffer, Friedemann Geiger, Robert Wolff, Karoline Weik, Fueloep Scheibler

**Affiliations:** 1 SHARE TO CARE (S2C) Team, National Competency Center for Shared Decision Making, University Hospital Schleswig-Holstein (UKSH) - Campus Kiel, Arnold-Heller-Straße 3, 24105 Kiel, Germany; 2 SHARE TO CARE (S2C) GmbH, Cologne, Germany; 3 TAKEPART Media+Science GmbH, Cologne, Germany; 4 Kleijnen Systematic Reviews Ltd., York, UK

**Keywords:** Shared decision making (SDM), Evidence-based Patient Decision Aid (PtDA), Evidence review, Evidence summarization

## Abstract

**Background:**

Recent publications reveal shortcomings in evidence review and summarization methods for patient decision aids. In the large-scale “Share to Care (S2C)” Shared Decision Making (SDM) project at the University Hospital Kiel, Germany, one of 4 SDM interventions was to develop up to 80 decision aids for patients. Best available evidence on the treatments’ impact on patient-relevant outcomes was systematically appraised to feed this information into the decision aids. Aims of this paper were to (1) describe how PtDAs are developed and how S2C evidence reviews for each PtDA are conducted, (2) appraise the quality of the best available evidence identified and (3) identify challenges associated with identified evidence.

**Methods:**

The quality of the identified evidence was assessed based on GRADE quality criteria and categorized into high-, moderate-, low-, very low-quality evidence. Evidence appraisal was conducted across all outcomes assessed in an evidence review and for specific groups of outcomes, namely mortality, morbidity, quality of life, and treatment harms. Challenges in evidence interpretation and summarization resulting from the characteristics of decision aids and the type and quality of evidence are identified and discussed.

**Results:**

Evidence reviews assessed on average 25 systematic reviews/guidelines/studies and took about 3 months to be completed. Despite rigorous review processes, nearly 70% of outcome-specific information derived for decision aids was based on low-quality and mostly on non-directly comparative evidence. Evidence on quality of life and harms was often not provided or not in sufficient form/detail. Challenges in evidence interpretation for use in decision aids resulted from, e.g., a lack of directly comparative evidence or the existence of very heterogeneous evidence for the diverse treatments being compared.

**Conclusions:**

Evidence reviews in this project were carefully conducted and summarized. However, the evidence identified for our decision aids was indeed a “scattered landscape” and often poor quality. Facing a high prevalence of low-quality, non-directly comparative evidence for treatment alternatives doesn’t mean it is not necessary to choose an evidence-based approach to inform patients. While there is an urgent need for high quality comparative trials, best available evidence nevertheless has to be appraised and transparently communicated to patients.

**Supplementary Information:**

The online version contains supplementary material available at 10.1186/s12911-022-01777-x.

## Background

German legislation with the Patients’ Rights Act in 2013 as well as recent trends to involve patients more actively at a micro health care level imply that physicians and patients should follow Shared Decision Making (SDM) communication rules in preference-sensitive treatment situations [1–3]. This includes but is not limited to the requirement that physicians have to comprehensively inform their patients about the pros and cons of relevant treatment alternatives (§630e German civil law book [1]). This process can well be supported by instruments such as evidence-based Patient Decision Aids (PtDAs). In line with the definition of the International Patient Decision Aids Standards (IPDAS) Collaboration and as summarized by Stacey et al. 2017 [4, 5], PtDAs are understood as “Interventions that support patients to make decisions, by making decisions explicit, providing information about options and associated benefits/harms, and helping clarify congruence between decisions and personal values”. In line with this definition of PtDAs we define SDM as an approach where clinicians and patients share the best available evidence on treatment alternatives, discuss individual experiences and the patients’ situation, explore the patients’ preferences regarding treatment alternatives and where finally the patient gets to an informed decision [6].

This publication results from the “Share to Care (S2C)” project conducted at the University Hospital Medical Center Schleswig Holstein (UKSH), Campus Kiel, in Germany from 2017 to 2021 [7]. The S2C project was funded by a German government grant, the Innovation Fund, which aims to support the implementation of innovative concepts to improve patient care in Germany. S2C was designed to prove that multiple SDM interventions can be implemented into a busy hospital setting within a 4-year period. The main components of the S2C project are (1) to train the hospital physicians in SDM communication skills with their patients (2) to develop evidence-based PtDAs to support patients’ decision-making (3) to train health care professionals in the hospital as so-called decision coaches supporting patients in making decisions and (4) to promote patients’ active involvement in decisions. Details on these have been published in a study protocol [7].

In this paper we describe the second component of this large scale SDM-project and how it was “filled with life” in some more detail: the development of PtDAs to support patient decision making, provide evidence-based information on the pros and cons of treatment alternatives to patients and facilitate better SDM communication between physician and patient including the exchange of experiences, values and preferences.

In our PtDA development process, the International Patient Decision Aids Standards (IPDAS) [8–10] were consistently and carefully observed. A recent review of IPDAS criteria by Hoffman et al. 2021 on the domain of “basing the information in decision aids on comprehensive, critically appraised, and up-to-date syntheses of the evidence’’ included a review of the evidence base of 471 decision aids [11]. This review revealed that 33% of PtDAs did not report any references to the scientific evidence used, despite being recommended by IPDAS. Of the remaining ones, 33% cited at least one guideline, 44% reported at least one systematic review, and 23% cited at least one RCT. Only 14% reported methods to find and include evidence, and only 14% reported on evidence quality. These findings are in line with those by Dannenberg et al. 2018 [12], reporting that less than half of PtDA developers documented their approach to summarizing evidence [12]. Moreover, researchers in the field have recently been requesting more standardized review and evidence summarization processes for the development of PtDAs [12–14]. Also, the recent revisions to the IPDAS “quality criteria” on the inclusion of evidence in PtDAs state, e.g., that in the absence of high-quality systematic reviews, best available evidence should be searched for and appraised instead [11, 15].

In this paper, our aims were to (1) describe how evidence-based PtDAs are developed and how S2C evidence reviews for each PtDA are conducted in this large SDM implementation project (2) appraise the quality of the best available evidence identified in S2C evidence reviews for 71 PtDAs and (3) identify challenges associated with the identified evidence.

## Methods

### PtDA development and how S2C evidence reviews are conducted for each PtDA

Each PtDA followed the same development process, which is depicted in this section. This process was developed in the first 6–8 months of the S2C project time. It closely adheres to the IPDAS criteria [10] aiming at identifying sound and comprehensive evidence on treatment alternatives on the one hand, and at providing this information to patients in a balanced and unbiased manner in the PtDAs, on the other. Involving PtDA users throughout PtDA development—primarily patients and physicians—was considered a corner stone of project implementation from the beginning (also see protocol publication [7]). Each S2C evidence review conducted for PtDA development equally followed a standard process as also depicted in this section. Each review was conducted along the standards of evidence-based Medicine (EbM).

### Baseline characteristics of PtDAs and respective evidence reviews

To better understand for which types of PtDAs S2C evidence reviews were conducted, we describe the baseline characteristics of so far completed PtDAs in the S2C project (n = 71). Firstly, we assessed the type of treatment alternatives compared in the PtDAs including surgeries, non-drug interventions, drug treatments or a do-nothing alternative. Secondly, we addressed whether a real do-nothing alternative was included or an extended do-nothing alternative like, e.g., continue with treatment as before, chose best supportive/palliative care, or watchful waiting. Thirdly, we summarized how often we could use existing evidence reviews as primary source of evidence instead of doing a de novo systematic review. In specific, we accepted reviews that were conducted, e.g., for the development of evidence and consensus-based German treatment guidelines, which apply sound review methods and define treatment standards for physicians in Germany.

Finally, we describe features of the 71 completed evidence reviews such as average time needed to conduct such a review, average number of references reviewed and average number of times additional data analyses or searches were conducted since data from identified evidence was incomplete or insufficient.

### Appraising the quality of evidence identified in evidence reviews

We critically appraised the quality of the best available evidence used in the 71 PtDAs. We did Cochrane risk of bias assessments and applied the GRADE approach to rate the certainty of the body of evidence [16, 17]. The quality categories for the identified evidence were:

1. Mostly high-/moderate-quality evidence identified high confidence in findings. This category was chosen if primarily systematic reviews/meta-analyses of good quality RCTs or several such RCTs were identified to be sufficiently reliable to be used as primary data source.
2. Some fair amount of higher-quality evidence identified but also lower quality moderate confidence in findings. This category was chosen if systematic reviews/meta-analyses of other well-done studies were identified or/and one or several RCTs that appeared sufficiently reliable
3. Mostly lower-quality evidence identified low confidence in findings. This category was chosen if mostly non-synthesized, non-RCT evidence was identified or data from one or more systematic reviews of lower quality studies or RCT(s) with limitations.
4. Mostly very low-quality evidence identified very low confidence in findings; information mostly based on very low-quality studies, expert opinion, or physician advice, or, e.g., only a consensus-based guideline was available.

Apart from the overall quality appraisal of each evidence review, we also appraised the quality of the evidence supporting outcome-specific information. Finally, we specified whether the quality of evidence facilitated quantitative or qualitative statements to be included in the PtDA.

### Highlighting challenges related to identified evidence and evidence quality

While our evidence reviews followed standard processes, the identified evidence posed a number of challenges to us throughout the course of the project. We summarized these challenges to increase understanding of the issues that may arise from communicating best available evidence to patients.

## Results

### PtDA development and how S2C evidence reviews are conducted for each PtDA

**Fig. 1:**
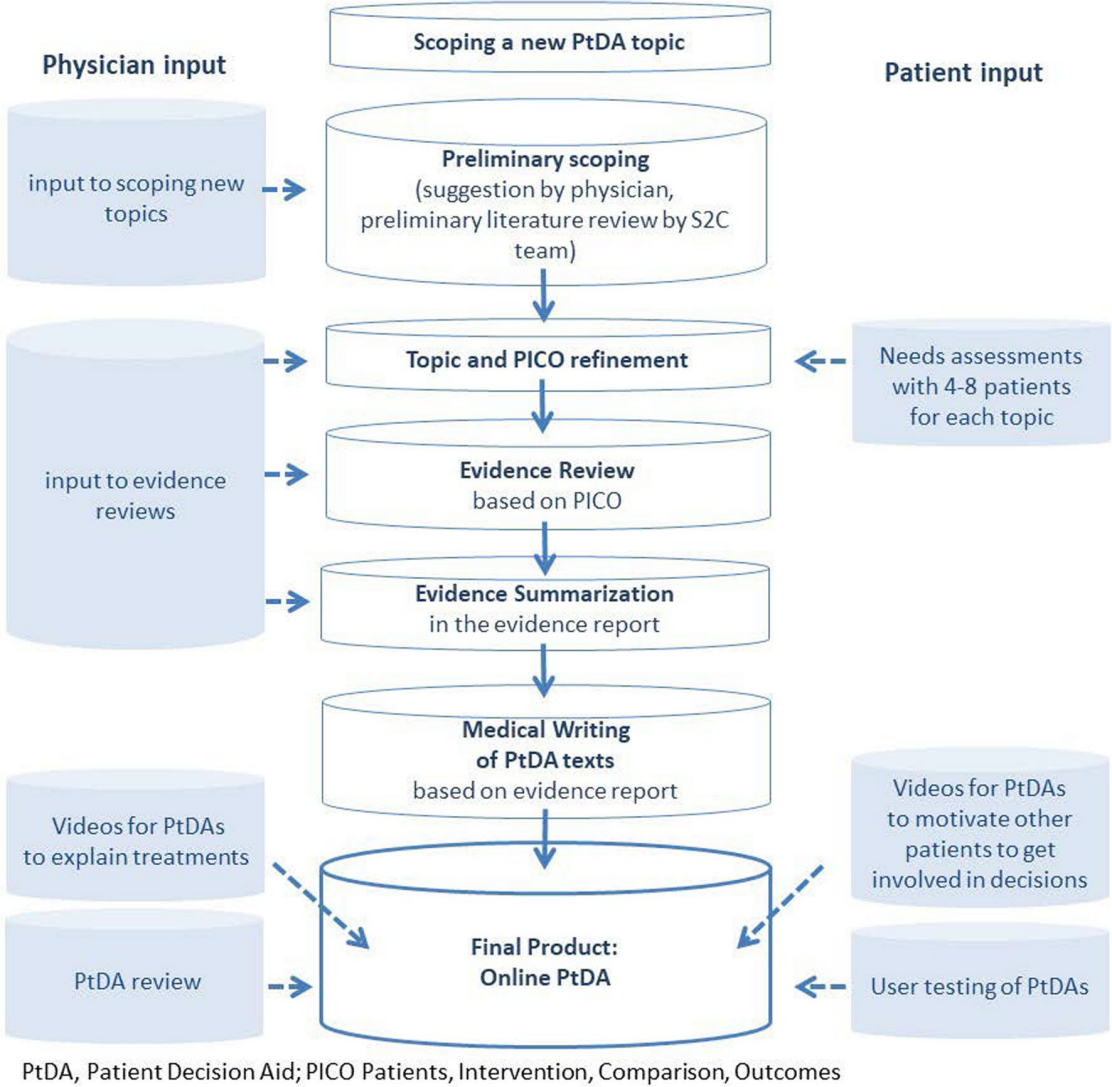
Process of online PtDA development in the S2C project

71 online PtDAs have been developed so far (Additional file 1). Each PtDA followed the development process depicted in Fig. 1.

Clinical departments of the hospital were successively enrolled in the project. Given the importance of involving users in decision aid development [18, 19], new topics for PtDAS were identified together with physicians and further developed with physician and patient input.

As displayed in Fig. 1, each of the PtDAs started with scoping a new topic together with physicians in the respective clinical department (“SCOPING a new topic” in Fig. 1). Criteria for topic selection were clinical and patient relevance as well as frequency of a decision to be taken. A preliminary literature review was conducted after first topic selection. Patients and physicians were then involved in refining the PICO for the respective topic (“PICO refinement” in Fig. 1) A PICO defines the target population of a PtDA (P), relevant treatment alternatives (intervention(s), comparator(s); I/C), and the outcomes of treatment (O). Patients were then involved via needs assessments, i.e., interviews were conducted with about 4–8 patients for each topic to explore their information and decision needs and, e.g., identify patient relevant treatment outcomes. Physicians were involved in scoping discussions with the S2C team.

Based on final topic scoping, the best available evidence was systematically searched for and appraised (“Evidence Review” in Fig. 1). Evidence reviews were conducted by one of two evidence review groups: Kleijnen Systematic Reviews (UK) and EBSCO Information Services (USA). Each S2C evidence review followed the same steps: (1) systematic search for evidence [20] including systematic reviews of Randomized Controlled Trials (RCTs) in the first place and lower level evidence such as individual RCTs, prospective studies and reviews of non-RCT in the second place; (2) systematic documentation of included reviews/studies for each outcome, (3) documentation of study quality (i.e., risk of bias and Grading of Recommendations Assessment, Development and Evaluation (GRADE) quality criteria assessment with up/downgrades for (in)directness, (im)precision, or inconsistency of findings (4) data extraction for each outcome including absolute effect estimates wherever possible, (5) summarization of these steps in an evidence report (“Evidence Summarization” in Fig. 1).

Best practice risk communication rules were followed when transferring findings from the evidence review to the texts for the PtDA [8, 21] (“Medical Writing of PtDA Texts” in Fig. 1). Evidence on outcomes such as mortality/survival, morbidity/symptoms, health related quality of life (HRQoL) or harms in all PtDAs are reported as answers to so-called Frequently Asked (Patient) Questions (FAQs). For example, for mortality the question answered is “Will the treatment help prolong my life?”, and for morbidity, the question answered is “Will the treatment decrease my symptoms?”.

Evidence reports are attached to each online PtDA as source of evidence. The final online PtDAs (“Final Product: Online PtDAs” in Fig. 1) consist of evidence-based texts informing about treatment alternatives, videos with physicians explaining treatment process to patients, videos with patients to motivate other patients to get involved in the respective decision, as well as a preference-elicitation tool (for further detail see an example decision aid here: Entscheidungshilfe zur Corona-Impfung).

In a final step, PtDAs are reviewed not only by the involved physicians but also by external reviewers as well as user-tested by 5–10 patients, respectively.

### Baseline characteristics of PtDAs and respective evidence reviews

A complete list of appraised PtDAs and included treatment alternatives is provided in Additional file 1. Baseline characteristics of PtDAs and their evidence reviews are displayed in Table 1. Most PtDAs (about 50%) in this project comprised comparisons between surgeries and other non-drug interventions. 20% of decisions involved comparisons between drug treatments only and 15% between non-drug interventions and drug treatments. About 15% were complex decisions between non-drug interventions including surgeries as well as drug treatments. About one third of PtDA included some kind of do-nothing alternative, of which the majority were extended do-nothing alternatives that involved some kind of non-curative treatment like best supportive/palliative care or active surveillance.

**Table 1:** Baseline characteristics of 71 PtDAs

	n	(%)
**Types of treatments being compared**		
Surgeries compared to other non-drug interventions^a^	19	27
Non-drug interventions/surgeries compared to each other	16	22
Drug treatments compared to each other	14	20
Drug treatments compared to non-drug interventions/surgeries	11	15
More than two different interventions types in comparison	11	15
**Do-nothing alternative (DNA) included or not**		
Yes	8	11
No	50	70
Extended DNA^b^ included	13	18
**Evidence base of comparisons**		
De-novo evidence review (evidence report)	48	68
Update evidence review plus guideline (update report)	11	15
Evidence & consensus-based German Clinical Practice Guideline	9	13
No reliable evidence available, lower level guideline or other kind of information was used	3	4

BSC, best supportive care; DNA, do-nothing-alternative; PtDA, Evidence-based Patient Decision Aid

^a^Includes 2 comparisons of surgery to a real do-nothing-alternative (DNA)

^b^Includes choice for best supportive care (BSC), palliative care, watchful waiting/active monitoring or stay on (drug) treatment as before

9 topics (13%) were entirely based on an already existing high-quality evidence review, e.g., from German evidence- and consensus-based clinical practice guideline. For the majority of PtDAs (59, 83%), a de-novo review or an update review was conducted. 4% of the 71 PtDAs were based on low-quality consensus-guidelines in combination with expert opinion or non-systematic clinical reviews due to a lack of available higher quality evidence sources.

On average, each of the S2C reviews took about 12 weeks to be completed. Each evidence review resulted in an evidence report attached to the PtDA. De-novo reports included on average about 25 references (ranging from 5 to 50 references), including guidelines, systematic reviews/meta-analyses and primary studies.

In about 10% of S2C evidence reviews pooled effect estimates were not readily available from the identified evidence but were self-calculated. In another 10% of S2C reviews, absolute numbers were not reported in studies/reviews but self-calculated. For example, these calculations were conducted by using baseline risks for one (control/comparison) group and applying risk ratios for the other group(s) to these baseline rates (for method of calculation see Institute for Quality and Efficiency in Healthcare (IQWiG) [22]). Finally, in about 5% of S2C evidence reviews no data on outcomes considered important by patients (as elicited in needs assessments) and/or by physicians could be identified in first round searches. Hence, second or third round evidence searches were conducted.

### Appraising the quality of evidence identified in evidence reviews

**Fig. 2:**
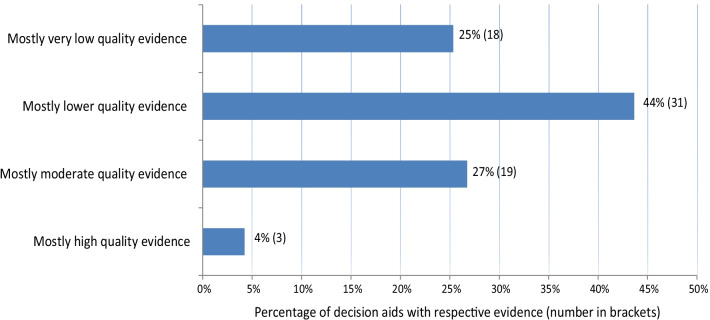
Overall quality of evidence in S2C reviews

We appraised the quality of evidence in all evidence-reviews. Main results are displayed in Fig. 2.

Based on the categories of evidence quality, about 30% of the 71 PtDAs were based primarily on overall high- or moderate-quality evidence. The majority were based on lower quality evidence. Of those based on high- or moderate-quality evidence about 1/3 were comparisons between drug treatments. Even if evidence was based on RCTs, about 40% of these were downgraded due to, e.g., certain risks of bias, indirectness or inconsistency.

The quality of evidence differed considerably for the different FAQs. 61 of the 71 PtDAs (86%) addressed morbidity-related outcomes, followed by 53 PtDAs (75%) addressing HRQoL and 33 (46%) addressing mortality/survival (Table 2). Outcomes that were not mortality, morbidity or HRQoL were counted in a separate category (called “other” in Table 2, included e.g., outcomes like weight loss or smoking cessation). While up to 50% of PtDA evidence reviews identified moderate to high quality evidence for the FAQ dimensions mortality and morbidity, only 30% of evidence on HRQoL was high or moderate quality. In about 60% of PtDAs no evidence on HRQoL was reported or it was too unspecific or uncertain to provide information to patients. Thus, in these PtDAs the potential impact of the treatment on HRQoL was often indirectly derived from data on morbidity and side effects/complications.

**Table 2:** Appraisal of outcome-specific evidence quality in 71 evidence-based PtDAs

Effectiveness outcomes	Mortality n (%)	Morbidity n (%)	HRQoL n (%)	Other n (%)	
PtDA with respective outcomes	33 (46%)	61 (86%)	53 (75%)	16 (23%)	
*Quality of evidence*					
High or moderate quevidence	15 (45%)	31 (51%)	17 (32%)	6 (38%)	
Low or very low evidence	18 (55%)	30 (49%)	36 (68%)	10 (62%)	

Harm outcomes	Side effects short term n (%)	Side effects long term n (%)	Complications short term n (%)	Complications long term n (%)	Treatment discontinuation n (%)
PtDAS with respective outcomes	29 (41%)	11(15%)	49 (69%)	32 (45%)	3 (4%)
*Quality of evidence*					
High or moderate evidence	19 (66%)	7 (64%)	20 (40%)	16 (50%)	2 (67%)
Low or very low evidence	10 (34%)	4 (36%)	29 (60%)	16 (50%)	1 (33%)

PtDA, Evidence based Patient Decision Aid

While more than 60% of the evidence for side effects (resulting mostly from drug treatments) was moderate or high, mostly low-quality evidence was identified for complications following non-drug interventions/surgeries (Table 2).

When transferring the evidence on effectiveness or harm outcomes to PtDAs, most of the information (about 70–80%) based on high or moderate level evidence could be quantified. For the remaining ones, no quantification was possible. This was often due to data being not or inconsistently reported. While for different HRQoL instruments between-group differences in specific scores were reported, these were often difficult to interpret. This primarily resulted from differing underlying HRQoL instruments/scales and a lack of information and analyses regarding whether the reported differences—even if statistically significant—were clinically meaningful or could somehow be used to define response criteria.

For effectiveness outcomes, between-group effects from direct comparative studies were available for at least half of PtDAs (for at least two alternatives included). For harms outcomes, between-group effects from direct comparative studies were available for about 20% of PtDAs (for at least two alternatives included). Due to the variety of included alternatives in PtDAs most of the data for treatment alternatives, however, originated from indirect comparisons.

### Highlighting challenges related to identified evidence and evidence quality

Challenges are summarized in Table 3. Overall, these challenges resulted from best available evidence needing to be summarized in a methodologically and clinically correct manner and at the same time be in a format to be easily understood by patients. We dealt with these challenges in an attempt to satisfy patients’ health informational needs in an as transparent and balanced as possible way. For example, we: (1) provided absolute numbers wherever possible (or self-calculated these numbers from the evidence if not readily available) for effectiveness and harm outcomes for all treatment options and put numbers (or lack of numbers) in context where needed (in qualitative terms) (2) provided indication as to the quality of evidence supporting the information and the confidence that is placed in the information, (3) provided a qualitative assessments of effectiveness/harm if quantification is not possible, (4) made early PtDA-specific decisions on how to frame outcomes best (e.g. mortality or survival information) in evidence review and summarization to provide most appropriate information to patients in a specific decision situation, (5) provided ranges of absolute effect when comparing alternatives lacking proof of significant or clinically relevant difference or limited to heterogeneous or uncertain evidence, (6) interpreted indirect, highly uncertain or heterogeneous evidence together with clinicians to make sure the information provided fits the population/subgroup of interest, (7) always reflected the evidence but also took into account clinical experience and expertise, (8) provided data on subgroups and outcomes as comprehensively as possible, taking resource and time-constraints of the project into account, (9) prioritized or did additional evidence searches for outcomes considered important to patients/physicians.

**Table 3:** Overview of challenges encountered most frequently in evidence reviews

Characteristics of the PtDA	Challenges related to available evidence
*Related to treatment alternatives*	
Very different treatment alternatives being compared (Extended) do-nothing compared to active treatments	No directly comparative evidence available: lack of evidence for one/some alternatives but comparative evidence for others Different absolute/relative estimates from heterogeneous reviews/studies Network meta-analysis not considered helpful if effect estimates different compared those of directly comparative evidence
Established treatments compared to innovations	Older versus newer evidence, absolute numbers differ: interpretation/transferability to current setting difficult Validity of estimates from older studies questionable
Treatments offered by competing clinical entities (e.g., cardio-surgeons vs. cardiologists) Specific clinical expertise with certain alternatives greater than with others (e.g., laparoscopic vs. open surgery)	Intense but productive discussions with clinicians on best available evidence/evidence interpretation Available evidence does not always seem to well reflect current clinical practice or clinical expertise at UKSH
*Related to target population, subgroups*	
Focus on e.g., elderly patients, children Effect modification/subgroups identified in evidence reviews	Transferability of results from evidence reviews to target group difficult, Support of clinicians needed to interpret evidence and its relevance for target group Need to provide relevant information for subgroups in the PtDA, e.g., for patients with diabetes No separate searches of additional evidence for identified subgroups were usually conducted
*Related to outcomes*	
Decision on framing of outcomes (e.g., mortality versus survival) Specific outcomes (effectiveness/harms) considered very important by patients or physicians	Outcomes reported in the evidence (mortality) were framed differently in the evidence summarization/PtDA (e.g., as survival) to provide most appropriate information to patients in specific situations Second/third round searches for evidence were conducted to fill data gaps

PICO, Patients, Intervention, Comparison, Outcomes; PtDA, Evidence-based Patient Decision Aid

## Discussion

### Main findings

This assessment of the evidence base of 71 decision aids for patients treated in a large University Hospital indicates that nearly 70% were—despite conducting a systematic and comprehensive literature review for each topic—based on rather low-quality and indirect evidence. Nevertheless, we consistently provided best available respective information to patients in our PtDAs.

Preference-sensitive decisions in a hospital involve very diverse treatment options. Best available evidence for these was often limited to non-RCT, non-directly comparative and likely biased studies. Also, while offering a do-nothing or wait-and-see alternative is recommended by IPDAS [8], this mostly was no realistic choice in this hospital-setting. Rather, extended do-nothing alternatives offering non-curative treatments like palliative care, best supportive care, or active surveillance were at choice.

About 30% of PtDAs were developed based on moderate or high-quality evidence. For mortality/survival outcomes, more than half was based on moderate to high quality, which likely will increase patients’ confidence in these highly patient-relevant findings. However, even RCT-based reviews or RCTs did not seem to be a guarantee for consistently high-quality evidence. RCTs frequently were downgraded due to biases like lack of blinding, the latter being most often an issue in RCTs comparing surgeries or surgeries to other non-drug interventions. In addition, RCTs often insufficiently reported on specific outcomes, especially HRQoL or harms, or used heterogeneous instruments/scales for outcome measurement, which complicated data interpretation and comparison across treatments.

### Comparison with other assessments of evidence reviews

On average, the quality of evidence in our reviews was lower than that reported in a recent assessment of Cochrane reviews by Howick et al. 2020 [23]. The latter reports that only a minority of outcomes for health care interventions are supported by high-quality evidence, which is in line with our findings. About 10% of Howick et al.’s identified evidence was high-quality (4% in our reviews), 37% was moderate-quality (27% in our reviews), 31% low-quality (44% in our reviews), and 22% very low-quality (25% in our reviews). However, our findings are not surprising in light of the project-specific issues discussed above. Moreover, while we assessed the average quality of evidence across outcomes, Howick et al. limited their assessment of evidence quality to the first-listed primary outcomes in Cochrane reviews, which are usually based on higher quality evidence than secondary endpoints like e.g., harm outcomes, which were part of quality assessment in our study.

Several recent reviews of published PtDAs suggest a lack of well documented evidence review and summarization processes [11, 12, 24]. In contrast, we consider our evidence reviews comprehensive and well documented. On the other hand, we clearly acknowledge the challenges identified by Zadro et al. [24], who states: “If only low-certainty evidence is available this could leave patients more uncertain than before they read the decision aid. … it is important to consider whether sufficient evidence exists to reasonably produce a well-balanced patient decision aid.” Dealing with often low-quality best available evidence resulting from a high-quality review of the evidence was indeed a huge challenge also in this project. However, instead of leaving patients without any information, or completely skip SDM in favor of a decision by the physician, the available evidence—in our view—should be presented to patients in a transparent way including information on evidence quality and confidence in findings. This is also in line with recent findings from an IPDAS review group [11]. We are uncertain whether we always succeeded in our attempt to transparently report on the pros and cons of treatment alternatives without causing patients to be more uncertain after reading our decision aid than before. However, we are convinced such communication is an important endeavor. Not providing information to patients is no solution. User testing of PtDAs in our project, which will be published separately in the near future, also do not indicate a systematic feeling of decision uncertainty or anxiety of patients after reading our decision aids.

Best ways of providing balanced information to patients—especially if based on low quality evidence—nevertheless is and will remain an area for research in many respects [25–27].

### Strengths and limitations

A major strength of our project is the IPDAS-compliant PtDA development with sound evidence review and summarization in combination with the size of the S2C project involving development of 71 decision aids. The latter facilitated to move to a more standardized approach to deal with the challenges summarized in Table 3. Such learning curves were also reported by other researchers, who developed several PtDAs in a relatively short period of time [28]. Another strength of our project is the involvement of physicians at all stages and of patients at key stages of the evidence review processes (Fig. 1), which also corresponds to the most recent IPDAS review recommendations [29, 30]. Olling et al. 2019 [31] even indicated that actively involving physicians in development processes may serve as a “Trojan horse” for introducing SDM to a new setting. Discussions with physicians throughout evidence production in this project were intense at times. However, in line with others [4, 29, 31], we feel that these discussions increased physicians’ trust in the carefully developed PtDAs.

A limitation may be that in this study we assessed results of our own S2C evidence reviews. However, since evidence reviews were conducted by independent external evidence review groups (Kleijnen Systematic Reviews, EBCO Information Services) based on the highest standards of evidence-based Medicine, we do not consider our assessment of the results being at high risk of bias. A major limitation of the S2C project itself, however, might be the development of PtDAs exclusively for a tertiary University hospital setting, which likely resulted in highly selected topics, mostly involving comparisons between surgeries or non-drug interventions. However, the hospital setting in Germany appears to be least familiar with SDM communication and treatment rules and therefore is in need of interventions such as the S2C program, which might have led to state funding of our project in Germany [32].

A real do-nothing alternative was usually not considered an option in this hospital setting. This setting likely explains many of the encountered challenges regarding low-quality—often non-RCT evidence—and the need for indirect comparison across treatments. A recent review of RCTs in surgery by Robinson et al. [33] concludes that more than half of the reviewed RCTs had some concern with bias and about 20% had high risk of bias—a finding we may confirm. Also, Robinson et al. report that about 80% of RCTs did not control for surgeon experience. The latter was also one of the identified challenges in this study (Table 3). We frequently discussed with physicians the outcome-specific results from our evidence reviews and how well these reflected their clinical experience. Taking physicians’ views into account on the one hand but sticking to the identified evidence on the other were key challenges. Such reality checks of evidence against physician experience, however, might be another argument for comprehensive user involvement in PtDA development as recently requested by an IPDAS working group [29].

## Conclusions

High standards for evidence review and summarization as part of PtDA development will likely make PtDAs more trustworthy and acceptable to patients and physicians. Evidence reviews and summarization in this project were carefully conducted. However, the evidence identified for our decision aids was indeed a “scattered landscape” and often of poor quality. Facing a high prevalence of low-quality, non-directly comparative evidence for treatment alternatives doesn’t mean it is not necessary to choose an evidence-based approach to inform patients. The experience from this large-scale PtDA development project shows: There is an urgent need for high quality comparative trials in many fields, however, in the meantime best available evidence has to be appraised and transparently communicated to patients in best possible ways.

## Supplementary Information

**Additional file 1.** List of patient decision aid topics in the Share to Care (S2C) project.

## Data Availability

The decision aids and evidence reviews generated in this project and the analyses of these described in this paper are available but restrictions apply to the availability of these data. Decision aids and reviews are currently restricted to use by patients and clinicians of the University Hospital Medical Center Schleswig Holstein (UKSH). Sample decision aids, reviews and the data analyses conducted for this project are available from the corresponding author on reasonable request and with permission of the UKSH and the sponsor of this study, the German Innovation Fund.

## Abbreviations

PtDA: Evidence-based Patient Decision Aid
FAQs: Frequently Asked Questions
GRADE: Grading of Recommendations Assessment, Development and Evaluation
HRQoL: Health-Related Quality of Life
HTA: Health Technology Assessment
IQWiG: Institute for Quality and Efficiency in Health Care
IPDAS: International Patient Decision Aids Standards
PICO: Patient, Intervention(s), Comparison(s), Outcome(s)
RCT: Randomized Controlled Trial
RoB: Risk of Bias
SDM: Shared Decision Making
S2C: Share to Care
UKSH: University Hospital Medical Center Schleswig Holstein
