# Supplementary material for: A scattered landscape: assessment of the evidence base for 71 patient decision aids developed in a hospital setting
Source: BMC Med Inform Decis Mak. 2022 Feb 17;22:44. doi: 10.1186/s12911-022-01777-x (PMC8855583; doi:10.1186/s12911-022-01777-x)
Supplement: Supplementary file 1 — Additional file 1. List of patient decision aid topics in the Share to Care (S2C) project. [file 12911_2022_1777_MOESM1_ESM.docx]

**Appendix 1. List of patient decision aid topics in the Share to Care (S2C) project**

| Topic / Name of decision aid | Treatment alternatives compared |
| --- | --- |
| **Department Departmentfor Internal Medicine IV: renal diseases and hypertension** | |
| (1) Chronic renal disease: kidney replacement therapy or conservative care? | hemodialysis, peritoneal dialysis, renal transplant, conservative/best supportive care |
| (2) Hypertension: how to prevent cardio-vascular diseases? | medical treatment, life-style changes |
| **Department for Neurology** | |
| (3) Epilepsy: which drug treatment? | different drug treatments in comparison |
| (4) Chronic remittent multiple sclerosis: how to start drug treatment? | different drug treatments in comparison |
| (5) Progressive Parkinson Disease – treatment with a pump or Deep Brain Stimulation (DBS)? | apomorphine pump, duodopa pump, DBS |
| (6) Asymptomatic Carotid Stenosis | optimized drug treatment, stent  (CAS = Carotid Artery Stenting), surgery (CEA = Carotid Endarectomy ) |
| (7) Symptomatic Carotid Stenosis | CAS, CEA |
| (8) Neuropathic pain | different drug treatments in comparison |
| **Clinic for Cardiology** | |
| (9) Coronary heart disease (CHD), Implantable cardioverter defibrillator (ICD): yes or no? | ICD, no ICD |
| (10) Cardiomyopathy, ICD: yes or no? | ICD, no ICD |
| (11) Atrial fibrillation: how to prevent stroke? | different anticoagulant treatments in comparison |
| (12) Symptomatic atrial fibrillation: catheter ablation or drug treatment alone? | catheter ablation (pulmonary vein ablation (PVI)), drug treatment |
| (13) CHD, three vessel disease: bypass or stent? | Percutaneous Coronary Intervention (Stent), bypass surgery (Coronary Artery Bypass Grafting) |
| (14) Aortic valve stenosis: TAVI or open surgery? | Transcatheter Aortic Valve Implantation (TAVI), Open surgery |
| (15) Decision for an artificial heart | Left Ventricular Assist Device (LVAD), no LVA but continued medical management |
| (16) Peripheral Arterial Disease –  conservative treatment or stent? | Percutaneous Transluminal Angioplasty (PTA) plus conservative treatment, conservative treatment alone |
| **Department for Neurosurgery** | |
| (17) Spinal stenosis: surgery, injections and/or conservative treatment? | surgery, epiudural injections, conservative treatment |
| (18) Herniated disc: surgery or conservative treatment | surgery, conservative treatment (including epidural injections) |
| **Department for Heart-Surgery** | |
| (19) CHD: which vessel for coronary bypass grafting? | breast artery, arm artery, leg vein |
| (20) Heart valve replacement: biological or mechanical? | biological heart valve, mechanical heart valve |
| (21) Abdominal aortic aneurysm: stent or open surgery? | stent, open surgery |
| **Department for Orthopedics, accident surgery** | |
| (22) Hip dysplasia: which potential treatment? | hip abduction braces, hip spica cast, traction, closed reduction, open reduction |
| (23) Hip Arthrosis: hip replacement or conservative management? | Total hip replacement (THP), conservative management |
| (24) Low arm fracture: which treatment? | closed reposition & cast, diferent types of fixation open reposition |
| (25) Implants: take out or not? | remove implant in a surgery, do not remove implant |
| (26) Knee Arthrosis: knee replacement or conservative management? | Total knee replacement (TKR), partial knee replacement (PKR), conservative management |
| (27) Cruciate ligament tear: surgery or conservative management? | surgery, conservative management |
| **Department for General Surgery (Visceral, Thoracical, transplant, pediatric surgery)** | |
| (28) Donate a kidney: yes or no? | kidney donation yes, no |
| (29) Obesity: conservative management and/or surgery? | conservative treatment (dietary/lifestyle intervention program), Roux-en-Y gastric bypass (RYGB), sleeve gastrectomy (SG), mini bypass |
| (30) Low rectal carcinoma: surgery with permanent stoma or not? | different surgery types followed by permanent stoma or not |
| (31) Low rectal carcinoma: neoadjuvant treatment plus surgery versus immediate surgery? | neoadjuvant radiotherapy, neoadjuvant radio-chemo-therapy, primary resection |
| **Department for Anaesthesia** | |
| (32) Non-specific chronic back pain: how to treat? | various drug- and non-drug treatments in comparison |
| **Department for Radiology** | |
| (33) Vestibularis Schwannoma: radiotherapy or surgery? | stent, surgery |
| (34) Early invasive breast cancer: radiotherapy - how best? | different modes of radiotherapy, no radiotherapy |
| **Department for Neuro-Surgery** | |
| (35) Incidental Intracranial Aneurysm: how to treat? | watchful waiting, active treatment (interventional or surgical) |
| (36) Essential tremor: how to treat? | DBS (Deep Brain Stimulation), MR (Magnetic Resonance)-guided focused ultrasound (MrgFUS) |
| **Department for Obstetrics and Gynaecology** | |
| (37) Hysterectomy in benign diseases: total or partial? | total hysterectomy, supra-cervical hysterectomy |
| (38) Hysterectomy in benign diseases: which surgical approach? | vaginal, laparoscopic, robotic-assisted laparoscopic hysterectomy |
| (39) Pelvic prolapse: which surgical treatment= | sacropexy, pectopexy, sacrospinal fixation |
| (40) Mamma reconstruction following mastectomy: how? | Implant above/below breast muscle |
| (41) Childbirth with breech presentation: caesarean section or not? | caesarean section, natural birth (including alternative treatments such as external cephalic version (ECV)) |
| (42) Endometriosis: which drug treatment could help? | different drug treatments in comparison |
| (43) Prenatal diagnostics: which tests? | prenatal screening measures according to German maternity recommendations |
| (44) Surgery of cervical carcinoma: open or laparoscopic? | laparoscopic surgery, open surgery |
| **Department for Internal Medicine I** | |
| (45) Severe asthma: which drug treatment? | different combination drug treatments in comparison |
| (46) Colectomy in colitis ulcerosa: when to do colectomy? | colectomy, no colectomy (but continued medical management) |
| (47) Colorectal cancer screening for women older than 50: yes or no? | coloscopy, stool test |
| (48) Colorectal cancer screening for men older than 50: yes or no? | coloscopy, stool test |
| (49)Colitis ulcerosa: treatment with biologicals yes or no? | different biological treatments in comparison |
| (50) Morbus Crohn: treatment with biologicals yes or no? | different biological treatments in comparison |
| (51) IPMN – surgery or active watchful waiting? | surgery, active surveillance/watchful waiting |
| (52) Stop smoking: how to? | different treatments (drugs and cognitive behavioral treatment/counselling) in comparison |
| (53) Hepatic cellular carcinoma in patients with cirrhosis: systemic treatment or ablation | systemic treatment, ablation |
| (54) Rheumatoid Arthritis I: options for conventional baseline treatment | different drug treatments in comparison (conventional anti-rheumatic drugs, corticosteroids) |
| (55) Rheumatoid Arthritis II: additional treatment options | different drug treatments in comparison (biologicals, Janus-Kinase-Inhibitors (JAK)) |
| (56) Get vaccinated against Corona: yes or no? | Vaccination / no vaccination |
| **Department for Internal Medicine II - Oncology** | |
| (57) Bone marrow transplant for myelodysplastic syndrome: yes or no? | allogeneic bone marrow transplant (BMT), no BMT |
| **Department for Oral and Maxillofacial Surgery** | |
| (58) Colum Fractures: surgery of conservative treatment | concervative treatment, surgery |
| **Department for Dermatology** | |
| (59) Moderate to severe atopic excema: which systemic treatment? | different drug treatments in comparison |
| (60) Moderate to severe psoriasis: conventional or biological treatment? | different conventional / biological drug treatments in comparison |
| (61) Actinic keratosis: which treatment? | different drug and non-drug interventions in comparison |
| (62) Allergene Immune Therapy: sublingual or injection treatment? | sublingual Immune Therapy (SLIT); Subcutaneous Immune Therapy (SKIT) |
| **Department for Pediatric cardiology** | |
| (63) Pulmonary Valve Replacement in children: surgery or catheter-based intervention? | surgery, catheter-intervention |
| (64) Prophylactic anticoagulation following Fontan-procedure: yes or no and which one? | anticoagulants, no anticoagulants |
| **Department for Pediatrics** | |
| (65) Juvenile Ideopathic Arthritis: glucosteroid injections or systemic treatment? | glucocorticoid treatment: systemic, injections |
| **Department for Conservative dentistry and paradontology** | |
| (66) Jaw injuries/diseases: endodontic treatment or tooth extraction? | endodontic treatment, extraction of teeth |
| **Department for Orthodontics** | |
| (67) Lack of space in the jaw: extract teeth or do endodontic treatment? | endodontic treatment, extraction of teeth |
| **Department for Dental Prosthetics** | |
| (68) How many implants in the toothless jaw? | implants, prothesis |
| **Department for Nuclear Medicine** | |
| (69) Morbus Basedow: radioiodine therapy or surgery for definite treatment? | radioiodine therapy, surgery |
| (70) Unifocal functional Autonomy: radioiodine therapy or surgery? | radioiodine therapy, surgery |
| **Department for Opthalmology** | |
| (71) Cataract surgery: now or later? | cataract Surgery, no cataract surgery |
